# P-338. Impact of an Educational Program on Hand Hygiene Practice and Healthcare-Associated Infections in a Tertiary Care Hospital in Rural South India

**DOI:** 10.1093/ofid/ofae631.540

**Published:** 2025-01-29

**Authors:** Jomel Raju, Maria Tom

**Affiliations:** St. Joseph's College of Pharmacy, Cherthala, Pala, Kerala, India; St. Joseph's College of Pharmacy, Cherthala, Pala, Kerala, India

## Abstract

**Background:**

Effective hand hygiene is pivotal in averting healthcare-associated infections (HAIs), especially in resource-limited settings. This study aimed to evaluate baseline hand hygiene practices among healthcare providers (HCPs) and the impact of an educational intervention on reducing HAIs in a rural South Indian tertiary care hospital.

**Figure:**
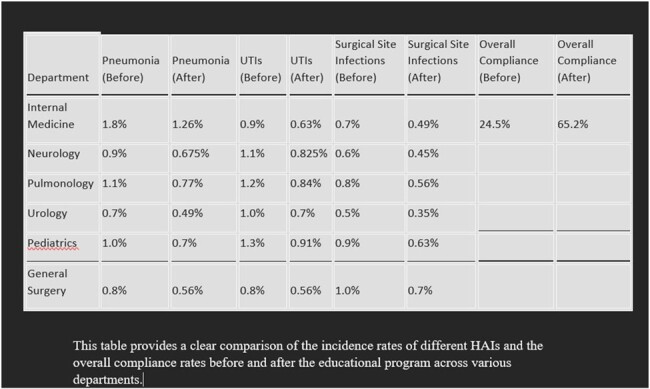

**Methods:**

Over a 12-week period, an observational study was conducted across internal medicine, neurology, pulmonology, urology, pediatrics, and general surgery departments. Hand hygiene compliance among HCPs was meticulously observed, while HAIs were monitored using CDC definitions. Subsequent to the baseline assessment, a targeted hand hygiene education program was initiated, focusing on enhancing compliance among 190 nurses.

**Results:**

A total of 1817 inpatients were enrolled in the study, with 55% male and 45% female, and age ranging from 1 to 90 years (median age: 50 years).

Among the 1817 inpatients, HAIs exhibited varying incidence rates across departments. Notably, pneumonia was the most prevalent HAI in the internal medicine department (incidence: 1.8%), while urinary tract infections were prominent in neurology and pulmonology (incidence: 0.9%), and surgical site infections in urology (incidence: 0.7%). Hand hygiene compliance varied across departments, with internal medicine demonstrating the highest compliance (24.5%). Post-implementation of the education program, compliance significantly improved to 65.2%. Consequently, there was a notable decrease in HAI rates, with pneumonia cases in internal medicine decreasing by 30%, and urinary tract infections in neurology and pulmonology decreasing by 25%.

**Conclusion:**

This study underscores the pivotal role of hand hygiene practices among HCPs in preventing HAIs, particularly in resource-limited settings. The implementation of targeted educational interventions resulted in a substantial improvement in compliance and a subsequent reduction in HAI incidence rates. These findings emphasize the ongoing need for concerted efforts to enhance hand hygiene practices and mitigate the risk of HAIs in rural healthcare settings. Further research and interventions are warranted to sustain these improvements and ensure optimal patient safety outcomes.

**Disclosures:**

**All Authors**: No reported disclosures

